# Automated information extraction from plant specimen labels using OCR and large language models

**DOI:** 10.3897/BDJ.14.e177202

**Published:** 2026-01-19

**Authors:** Juan Wen

**Affiliations:** 1 Kunming Institute of Botany, Chinese Academy of Sciences, Kunming, China Kunming Institute of Botany, Chinese Academy of Sciences Kunming China

**Keywords:** plant specimen digitization, text recognition, PaddleOCR, large language model, information extraction, artificial intelligence

## Abstract

The digitization of herbarium specimens is crucial for advancing biodiversity research and data sharing. However, this process is often hindered by the inefficiency of manual transcription and the technical challenges posed by the massive volume of specimens, heterogeneous label layouts, and the prevalence of handwritten texts. To overcome these bottlenecks, this study proposed an automated pipeline that integrates the PadddleOCR engine with the DeepSeek large language model (LLM) for structured information extraction from specimen labels.

The pipeline is designed to extract 16 key metadata fields from both printed and handwritten labels. Evaluated on a benchmark dataset, it achieved a high field-level accuracy of 95.4% for printed labels, demonstrating strong reliability. For handwritten labels, the system maintained functionality while correctly identifying its limitations through a confidence-based quality control mechanism. A key finding was the compensatory role of the LLM, which effectively corrected upstream OCR errors, as evidenced by a weak correlation (*r* = 0.32) between OCR (Optical Character Recognition) confidence and final extraction accuracy. This hybrid architecture ensures data security through local image processing and cost-efficiency via text-only LLM parsing.

This work provides a robust, scalable, and practical solution for accelerating the digitization of botanical collections. The method is directly applicable to real-world digitization workflows and promises to significantly enhance the efficiency of biodiversity data creation and sharing.

## Introduction

Plant specimens serve as vital records of species distribution, morphology, and ecological data, supporting research in taxonomy, biodiversity conservation, and climate change. Although preservation techniques have evolved from simple drying to standardized practices, traditional herbarium management remains challenging due to substantial spatial requirements, strict environmental controls, and vulnerability to physical degradation. In response, large-scale digitization has been initiated worldwide, bridging physical preservation with digital sustainability and driven by coordinated mass-digitization initiatives across natural history collection (Blagoderov and Smith 2012).

**Figure 1.**
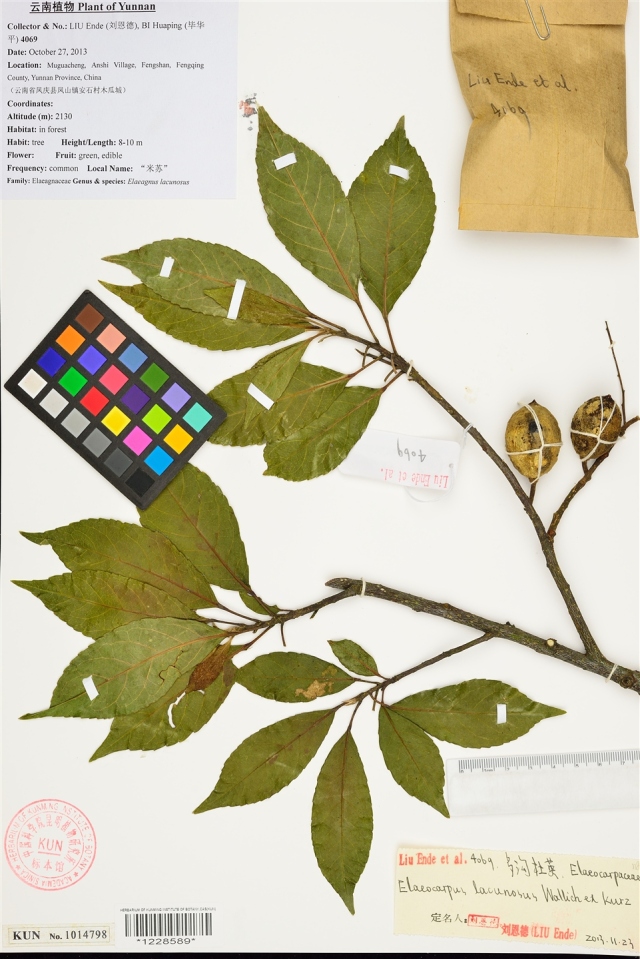
An exemplar digitized plant specimen illustrating key components such as the plant body, data label, and barcode. Source: Herbarium of Kunming Institute of Botany (KUN) via Chinese Virtual Herbarium (https://www.cvh.ac.cn/spms/detail.php?id=ea8860a5), licensed under CC BY-NC-ND 4.0.

A typical digitization workflow includes specimen selection, high-resolution imaging, post-processing, metadata entry, and integration into open-access databases (Kirchhoff et al. 2018, Novikov and Nachychko 2025). While automated, high-throughput imaging workflows have substantially accelerated image acquisition (Sweeney et al. 2018), converting physical specimens into structured digital data compliant with standards such as Darwin Core (Wieczorek et al. 2012) depends fundamentally on the information captured within specimen images. The resulting digitized specimen image generally consisted of the plant body, label information (core metadata), collection identifiers, and scale/color references, often supplemented with barcodes, annotations, or detached organs' envelopes (see Fig. 1).

Crucially, the information contained within these images—particularly on the specimen labels—is increasingly following structured formats encompassing taxonomic, geographic, collection, and ecological information. The growing standardization enables the application of computer vision (CV) and natural language processing (NLP) techniques to automatic digitization. However, label transcription remains highly labor- and time-intensive and constitutes a major bottleneck in specimen digitization; despite declining imaging costs, label data capture is still predominantly manual and can account for up to approximately 90% of the total digitization effort, thereby limiting throughput and consistency (Blagoderov et al. 2012, Nelson et al. 2012, Kirchhoff et al. 2018).

Optical Character Recognition (OCR) technology provides a promising alternative by extracting text from images into machine-encoded format (Mori et al. 1999). Driven by deep learning, modern OCR has evolved from rule-based methods to end-to-end neural architectures that combine CNNs (Convolutional Neural Networks), RNNs (Recurrent Neural Networks), and attention mechanisms for accurate recognition of complex or handwritten texts (Long et al. 2020, Shi et al. 2017, Shi et al. 2019). A prominent example is PaddleOCR, a state-of-the-art system developed by Baidu that supports over 80 languages and enables lightweight, cross-platform deployment (Du et al. 2020). Its versatility has been demonstrated across diverse domains, from license plate recognition to the digitization of historical documents (Liu and Liu 2024, Zhang and Wang 2024, Ma et al. 2024, Lu et al. 2024).

However, OCR alone is insufficient for generating structured metadata. Subsequent parsing of recognized text remains a challenge, and existing methods each present distinct limitations. Rule-based systems lack flexibility; dictionary-based methods depend on predefined lexicons; and layout-aware models struggle with the variable formats typical of specimen labels (Smith 2007, Tkaczyk et al. 2015, Heidorn and Wei 2008). Named entity recognition (NER) models, while effective in constrained settings, require extensive labeled data for training and remain sensitive to OCR noise and layout heterogeneity (Lample et al. 2016). Recent herbarium-focused studies applying OCR in combination with NER have demonstrated practical extraction pipelines but also reported substantial error propagation and pronounced field-level variability, particularly for heterogeneous label layouts and handwritten content (Takano et al. 2024).

In recent years, large language models (LLMs) have emerged as powerful tools for information extraction, offering robust semantic understanding and strong tolerance to noise and format variability. Unlike rule-based or NER-based approaches, LLMs can infer implicit field relationships, resolve semantic ambiguities, and generalize across heterogeneous layouts without requiring extensive task-specific annotation. These capabilities make LLMs particularly suitable for specimen label parsing, where fields are often inconsistently ordered, expressed using domain-specific aliases, and even implicitly structured.

In the context of herbarium digitization, LLMs provide a flexible complement to OCR by operating directly on noisy or partially recognized text outputs, which may help mitigate propagation of errors from upstream recognition stages. Recent advances in prompt-based information extraction further enable domain adaptation without retraining, supporting scalable deployment across collections with diverse label conventions.

To address the specific challenges of Chinese specimen labels—including heterogeneous layouts, mixed print–handwritten content, and frequent field alias variations—this study proposes a hybrid framework that integrates PaddleOCR with the DeepSeek LLM. Through domain-specific prompt design and a confidence-based filtering mechanism, the proposed approach enables accurate parsing of multi-line, parallel, and implicitly structured fields. By combining localized OCR processing with LLM-based semantic interpretation, the framework enhances digitization efficiency while maintaining data security, offering a practical and scalable solution for large-scale biodiversity data mobilization.

## Materials and Methods

**Data Sources**

Experimental data were sourced from the Chinese Virtual Herbarium (CVH), an authoritative platform for digitized specimen information provided by the National Plant Specimen Resource Center (http://www.cvh.ac.cn). All specimen images are licensed under a CC BY-NC-ND 4.0 license. To capture diverse label styles, we selected images from three of China's major herbaria: the Herbarium of the Institute of Botany (PE), the Kunming Institute of Botany (KUN), and the South China Botanical Garden (IBSC).

A total of 600 images were randomly sampled (200 per institution), comprising 120 printed and 80 handwritten label images from each, resulting in a combined dataset of 360 printed and 240 handwritten specimens. This 40% proportion of handwritten samples captures substantial real-world variability in writing styles, layouts, and image quality.

For evaluation purposes, a control subset of 240 images (80 per herbarium, with 48 printed and 32 handwritten) was manually annotated with 16 core metadata fields to serve as the gold standard. This control dataset specifically includes challenging cases such as irregular layouts and cursive handwriting to enable rigorous evaluation (Table 1).

**Research Environment Configuration**

The processing pipeline was implemented in Python 3.10. Text detection and recognition were performed using PaddleOCR with the PP-OCRv3 framework, while structured field extraction was handled through the DeepSeek LLM API. Experiments were conducted on a macOS system with an Apple M2 chip and 16 GB of unified memory.

**OCR and Structured Extraction Framework**

Chinese plant specimen labels present significant challenges for automatic processing due to diverse layouts, mixed printed and handwritten text, and multilingual content. To address these complexities, this study employed the PaddleOCR with the PP-OCRv3 configuration, which integrates a DB++ detector with ResNet18_vd backbone for text detection and an SVTR-LCNet recognizer with CTC decoding for text recognition. Text orientation correction was handled by the PP-OCRv2 classifier. This configuration provides an optimal balance between recognition accuracy and computational efficiency for heterogeneous specimen labels.

Following OCR processing, the recognition results were passed to the DeepSeek LLM API through a carefully designed prompt template for structured parsing. The system extracted 16 key metadata fields aligned with Darwin Core standards, including: recordNumber, eventDate, recordedBy, locality, longitude, latitude, elevation (m), habitat, associatedTaxa, habit, family, scientificName, vernacularName, identifiedBy, dateIdentified, and occurrenceRemarks (for complete definitions and mappings, see Suppl. material 1). All outputs were indexed by image filename and stored in structured JSON format for subsequent analysis.

**Domain-Specific Prompt Engineering**

To enhance extraction accuracy and adaptability, this study developed a set of domain-specific prompts for the DeepSeek LLM based on five core principles:

1. **Field Alias Normalization**: Standardizes synonymous terms for the 16 target metadata fields to ensure consistent mapping despite lexical variations in original labels.
2. **Field Segmentation**: Directs the model to split conjoined fields (e.g., “collector/collection number”) into discrete schema elements.
3. **Temporal Format Standardization**: Converts all date expressions to ISO format (“YYYY-MM-DD”) to ensure temporal consistency.
4. **Units and Symbol Unification**: Standardizes coordinate units to “°”, “′”, “″”, and specifies the Chinese dun hao (“、”) for list separation, converting other delimiters to reduce parsing errors.
5. **Contextual Association**: Leverages the LLM’s reasoning capability to interpret implicit relationships across text lines, enabling accurate parsing of multi-line descriptions and unlabeled fields.

**Integrated Processing and Evaluation Workflow**

**Figure 2.**
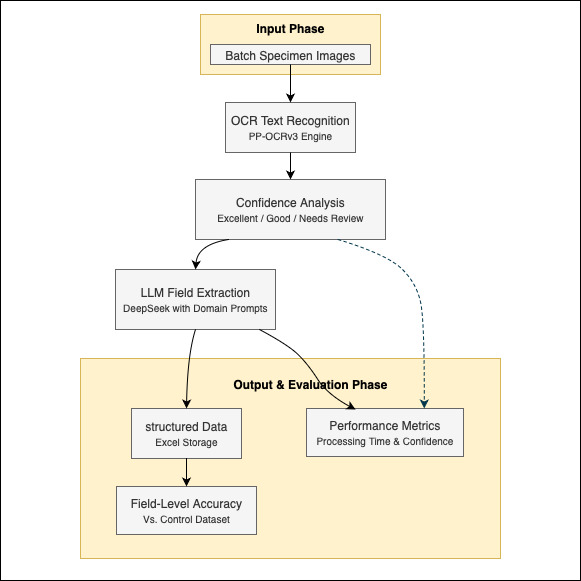
The workflow for specimen label information extraction and performance evaluation.

The automated pipeline integrates PaddleOCR for text recognition and DeepSeek for structured extraction (Fig. 2), processing specimens through the following stages:

1. **Batch Processing with Checkpointing**: Images are processed iteratively from a predefined directory, with a checkpointing mechanism that tracks processed filenames in the output Excel file to enable seamless resumption.
2. **OCR Text Recognition**: The PaddleOCR engine processed each image, outputting recognized text lines with corresponding confidence scores.
3. **Confidence-based Quality Tiering**: Each text line is categorized as “Excellent” (≥0.95), “Good” (0.9–0.95), or “Needs Review” (<0.9) based on OCR confidence scores.
4. **LLM-based Structured Extraction**: All text lines from an image are concatenated and submitted to the DeepSeek API with domain-specific prompts, returning structured data in JSON format.
5. **Incremental Result Storage**: Extracted data, OCR statistics and processing timestamps are immediately appended to a master Excel file, enforcing checkpointing and ensuring data persistence.
6. **Progressive Performance Monitoring**: Cumulative statistics (processed images, average processing times, and mean confidence) are updated throughout batch processing, with low-confidence (<0.9) images flagged for potential manual review.
7. **Field-level Accuracy Assessment**: Extraction results for the control dataset are compared against manual annotations with each field scored as 1 (correct), 0.5 (partially correct), or 0 (incorrect) for fine-grained accuracy evaluation.

## Results and Analysis

**Text Recognition Performance at the Image Level**

**Figure 3.**
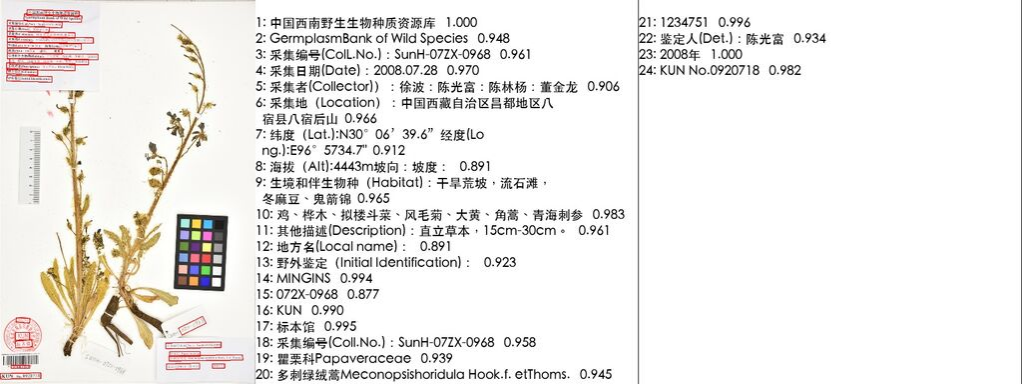
Visualization of PaddleOCR recognition results for a specimen image.

The PaddleOCR system was employed for automated text recognition on digitized plant specimen labels. For each processed image, the system output both textual content and a corresponding confidence score per recognized line, quantifying recognition certainty. As shown in Fig. 3, all text lines in a sample label were successfully identified. The first line, “中国西南野生生物种质资源库” (“Southwest China Wildlife Germplasm Bank”), was recognized with a confidence score of 1.000, and the average confidence across all lines reached 0.9536, indicating high overall recognition reliability.

To enhance quality control, a confidence threshold of 0.9 was applied to automatically flag text lines for manual inspection. In Figure 3, the 8th line “海拔（Alt):4443m坡向：坡度：” was flagged with a confidence of 0.891. Manual verification confirmed a minor punctuation error (a wrong bracket in “Alt”). This case demonstrates the practical value of confidence-based filtering: it not only identifies subtle inaccuracies but also efficiently prioritizes challenging recognitions for expert review, thereby optimizing verification workflows.

**Structured Field Extraction Performance**

Plant specimen labels exhibit substantial heterogeneity across institutions, time periods, and formatting conventions (see Suppl. material 2 for representative examples). Critical fields such as collection number appear under numerous aliases (e.g., “采集编号”, “采集号”, “Collect No”); dates follow inconsistent patterns (“1982年08月24日”, “8/10/2008”); and coordinates are expressed in diverse formats (“117.6213°E”, “E99°06′355″ N27°11′628″”). Additional complexities arise from line breaks within single fields, multiple fields presented on one line, and irregular use of punctuation and units, which collectively hinder traditional rule-based extraction.

To overcome these challenges, this study engineered a set of domain-specific prompting principles for the DeepSeek LLM, transforming the generic model into a specialized parser for herbarium labels. The core of our methodology lies not in building separate technical modules, but in encoding solutions directly into the prompt, which guides the LLM to perform alias normalization, temporal and unit standardization, field segmentation, and context-aware association.

This prompt-driven approach effectively handles the described complexities: it correctly segmented conjoined fields like “采集人/采集号” into discrete recordedBy and recordNumber values, normalized the date “1982年08月24日” to the ISO format “1982-08-24”, and successfully performed semantic inference to extract the scientificName even without an explicit field label. These results demonstrate that our prompting framework reliably equips a general-purpose LLM to perform accurate, structured information extraction from highly heterogeneous and complex specimen labels, achieving a level of adaptability that would be difficult to implement with conventional methods.

**Batch Processing Performance**

**Figure 4.**
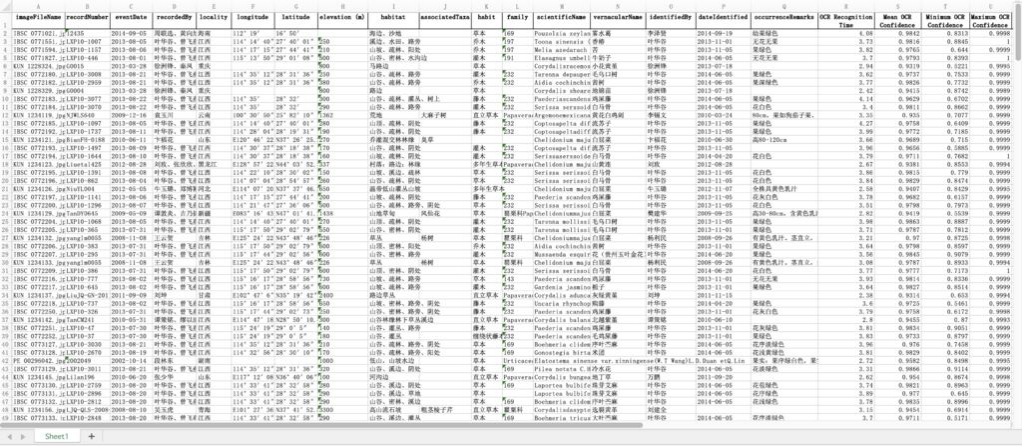
Batch output results for image text recognition.

An automated batch processing pipeline was implemented to evaluate the scalability and robustness of our method, integrating OCR recognition, confidence analysis, and LLM-based extraction with incremental checkpointing to ensure fault tolerance. Consolidated outputs were stored in a structured Excel file for analysis (Fig. 4, detailed results are provided in Suppl. material 3).

As summarized in Table 2, the system demonstrated high reliability on a set of 360 printed-label images. Excluding one outlier image that caused critical OCR failure and extracted no data due to poor image quality, the average OCR confidence for the remaining images (n=359) was 0.962 (±0.019). Among these, only a single image fell below the 0.9 threshold due to pronounced text skew, underscoring the consistency of recognition. In terms of efficiency, processing these images took 19.60 minutes (mean OCR time: 3.28 s/image) for OCR and 50.39 minutes (mean: 8.42 s/image) for DeepSeek extraction.

A marked performance difference was observed on 240 handwritten-label images, where factors like scribbled writing, ink fading, paper degradation, and poor imaging conditions reduced average OCR confidence to 0.891 (±0.029). Over half (55.4%, n=133) of these images were correctly flagged with confidence below 0.9, validating the effectiveness of the confidence-based filtering mechanism. Processing times were slightly lower (OCR: 14.08 min, 3.52 s/image; DeepSeek: 28.49 min, 7.12 s/image), likely due to fewer recognizable text lines per image.

These results confirm the pipeline’s robustness for printed specimen labels, providing a fast and reliable foundation for large-scale digitization. For handwritten labels, the system intelligently manages uncertainty by flagging low-confidence extracts, thereby effectively orchestrating a human-in-the-loop workflow.

**Field-Level Accuracy Evaluation**

Field-level extraction accuracy was evaluated against a manually annotated ground truth of 240 images (144 printed, 96 handwritten). Accuracy was calculated exclusively for fields present in the original label; for instance, geographical coordinates typically absent from historical handwritten specimens were excluded from calculations where they did not appear.

For printed label images, the pipeline achieved a high average field-level accuracy of 95.4% (Table 2), with 127 images (88.2%) scoring above 0.9. The low standard deviation (0.051) indicates consistent performance across diverse image conditions.

**Figure 5.**
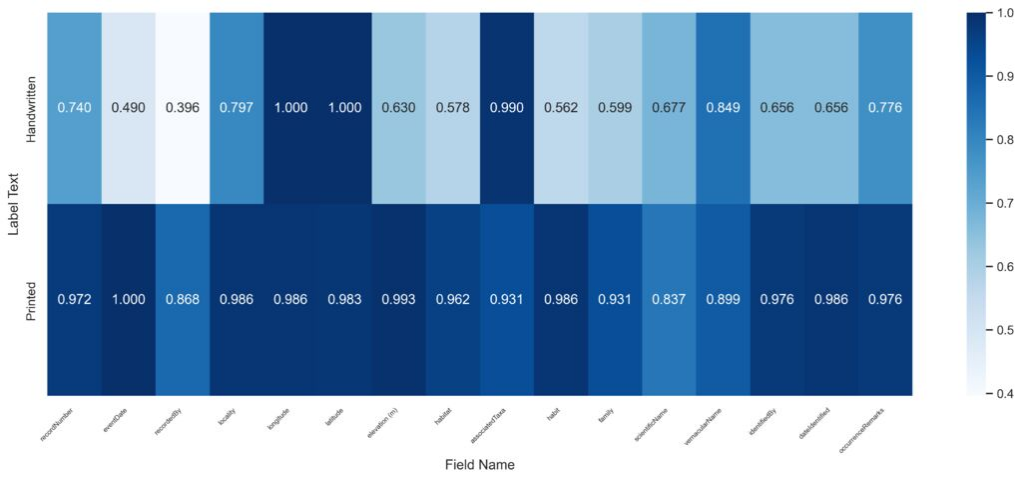
Heatmap of field-level recognition accuracy across printed and handwritten labels. **Note**: The longitude, latitude, and associatedTaxa fields were excluded from accuracy calculation for handwritten labels as they are typically absent from the original specimens.

Analysis of individual fields (Fig. 5) identified scientificName, recordedBy, and vernacularName as having relatively lower accuracies (0.837, 0.868 and 0.899, respectively), primarily attributable to OCR errors on low-quality image, which led to incomplete or misrecognized text. The remaining 13 fields consistently achieved accuracies above 0.9, demonstrating the method’s overall robustness.

**Figure 6.**
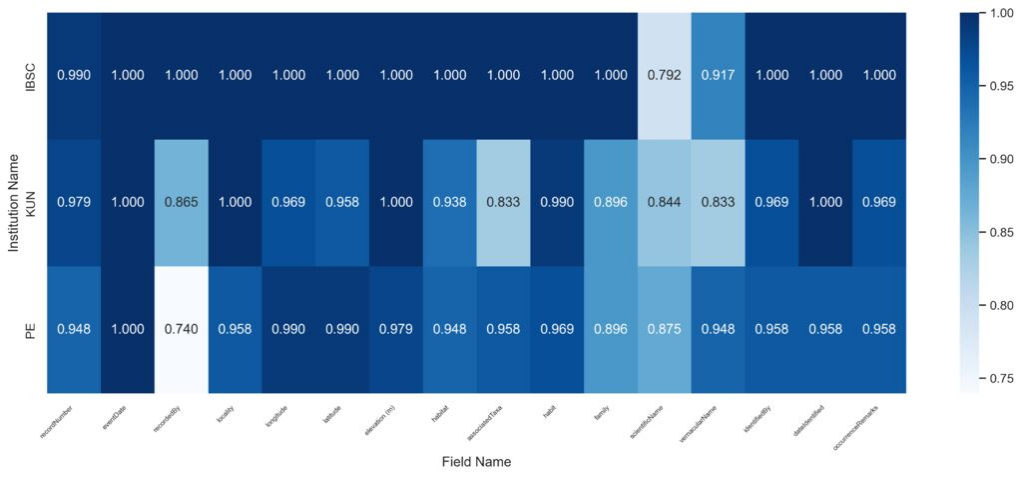
Heatmap of recognition accuracy by institution for printed labels

When grouped by herbarium (Fig. 6), the average accuracy for IBSC, PE, and KUN specimens was 0.981, 0.942, and 0.940, respectively. The observed variations, while modest, indicate that institution-specific label formats do influence the extraction outcome. This suggests that tailoring the prompt engineering to the conventions of each herbarium presents a promising avenue for achieving even higher accuracy.

**Figure 7.**
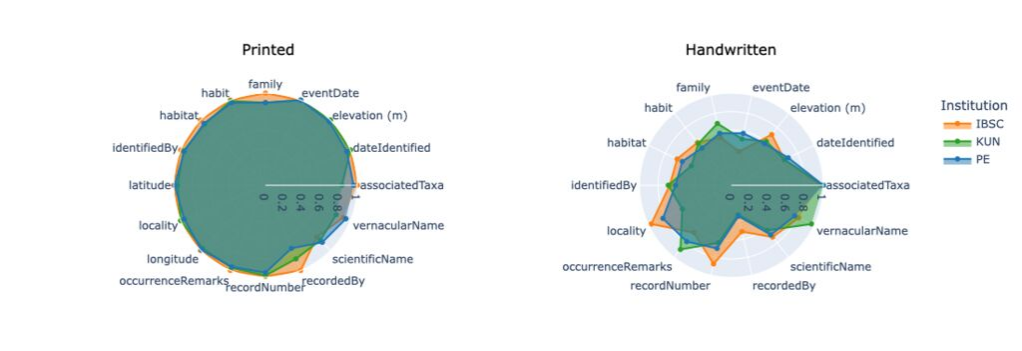
Radar charts of field-level accuracy for printed (left) and handwritten (right) labels

For handwritten labels, the overall field-level accuracy across 13 target fields was substantially lower at 64.7% (std=0.113). This reflects the compounded challenges of handwriting variability, image degradation, and frequent absence of key metadata fields (namely, longitude, latitude, and associatedTaxa) in historical specimens. The radar chart (Fig. 7) shows pronounced performance decline and higher heterogeneity across all fields, particularly for eventDate and recordedBy. Nevertheless, the fact that the system achieved successful recognition on neatly written fields confirms the pipeline’s potential applicability to less standardized scenarios when text legibility is adequate.

## Discussion

**Performance and Robustness of the OCR-LLM Pipeline**

This study developed an integrated OCR-LLM pipeline for automated information extraction from herbarium specimen labels. Quantitative evaluation demonstrated a high field-level accuracy of 95.4% on printed labels, underscoring the pipeline's robustness for standardized materials. In contrast, the performance on handwritten labels (64.7%) reflects the persistent challenges posed by historical specimens, including handwriting variability, image degradation, and inconsistent formatting. The pipeline’s ability to correctly flag over 55% of handwritten images for manual review further demonstrates its practical utility in real-world digitization scenarios where perfect accuracy is unattainable.

**The Compensatory Role of the LLM**

A key finding from our analysis is the weak but significant correlation between OCR confidence and final extraction accuracy for both printed (*r* = 0.324, *p* < 0.001) and handwritten (*r* = 0.281, *p* = 0.006) labels. This result challenges the conventional assumption that OCR confidence directly predicts end-to-end system performance. Instead, it reveals that the LLM acts as a compensatory module, effectively mitigating upstream OCR errors through contextual understanding and semantic reasoning. The system frequently compensates for character misrecognitions and punctuation errors, enabling high accuracy even from low-confidence OCR outputs. This decoupling is more pronounced for handwritten labels, where the LLM's ability to reconstruct fragmented text reduces the reliance on perfect optical input.

**Practical Implications for Digitization Workflows**

Our confidence-based quality control mechanism, combined with the LLM's error correction capability, provides a balanced approach to manage digitization quality. Although confidence filtering effectively identifies potential problem areas, the LLM ensures that many OCR errors don't propagate to final outputs. To maximize performance, we recommend coupling this tool with standardized imaging protocols (e.g., uniform lighting, high resolution) and institutional efforts to harmonize label formats. Furthermore, the observed variations in accuracy across herbarium, while modest, suggest that institution-specific prompt customization could yield additional improvements for large-scale digitization projects.

**Interoperability and Broader Impact**

The pipeline's outputs are structured to align with the Darwin Core standard, ensuring seamless integration with global biodiversity data infrastructures like GBIF (the Global Biodiversity Information Facility) and CVH. This interoperability is crucial for enhancing the findability, accessibility, and reusability of digitized specimen data. By transforming heterogeneous label information into standardized, structured formats, this approach significantly reduces the manual effort required for biodiversity data mobilization and enables more efficient reuse of specimen information across research domains.

## Conclusion

This study presents a robust, automated pipeline that synergistically combines PaddleOCR for text recognition and a Large Language Model (DeepSeek) for structured information extraction from herbarium labels. This hybrid architecture was deliberately chosen over end-to-end multimodal models to ensure data security (via local image processing), cost-effectiveness (using lightweight text APIs), and operational transparency (enabled by interpretable OCR confidence scores). Evaluation on 600 specimen images demonstrated the pipeline's effectiveness, particularly for printed labels where it achieved 95.4% field-level accuracy.

The core innovation lies in the demonstrated efficacy of the OCR-LLM collaboration mechanism. The weak correlation between OCR confidence and final accuracy confirms that the LLM serves a critical compensatory function, correcting upstream recognition errors through semantic understanding. This makes the pipeline particularly valuable for processing diverse and non-standardized collections where perfect OCR performance is unrealistic.

Future work will focus on enhancing performance on handwritten labels through domain-adapted OCR models and specialized image preprocessing techniques. Implementing a human-in-the-loop verification system, intelligently triggered by confidence scores, will further optimize the balance between automation and data quality. Exploring institution-specific prompt tuning represents another promising direction for improving accuracy across heterogeneous collections.

In summary, this pipeline offers a practical, secure, and efficient solution for transforming physical specimen labels into structured, sharable data. It addresses a critical bottleneck in herbarium digitization and holds significant promise for curators, biodiversity researchers, and data aggregators participating in the global effort to preserve and utilize our botanical heritage.

## Availability of data and materials

The original specimen images used in this study are publicly accessible through the Chinese Virtual Herbarium (CVH) platform. The benchmark dataset and manually annotated ground truth for the 240 control images, the demonstrative code for the OCR-LLM pipeline, along with installation and usage instructions, are available in the following GitHub repository: https://github.com/JW135/herbarium-OCR-LLM-pipeline.git.

The open-source OCR engine PaddleOCR is publicly available at: https://github.com/PaddlePaddle/PaddleOCR

The large language model API used in this work is provided by DeepSeek and is accessible via: https://www.deepseek.com/

## Supplementary Material

4DE140E0-FADE-5F39-9429-AAD11BFB4A0B 10.3897/BDJ.14.e177202.suppl1 Supplementary material 1 Darwin Core Field Mapping and Descriptions Data type image Brief description The following table details the 16 key metadata fields extracted by the pipeline, their formal definitions according to the Darwin Core standard, and representative examples of the Chinese label aliases that were normalized to each field. File: oo_1458097.png https://binary.pensoft.net/file/1458097 Juan Wen

8F669AA7-0DDA-53B2-AFEE-25EE8D6CBB4E 10.3897/BDJ.14.e177202.suppl2 Supplementary material 2 Representative Examples of Label Heterogeneity and Parsing Challenges Data type images Brief description This material provides visual evidence of the principal challenges encountered in the automated information extraction from herbarium specimen labels. The referenced images (a-p) illustrate the four major categories of heterogeneity that our OCR-LLM pipeline is designed to address.
1. Proliferation of Field Aliases
A single conceptual metadata field frequently appears under multiple, inconsistent labels, requiring a process of alias normalization for data consolidation.
• recordedNumber: Labeled as “采集号(Coll. No.)” (a), “采集编号(Col.No.)” (b), “采集编号” (c), “COLL. No.” (f), etc.
• locality: Identified as “采集地(Locality)” (a), “采集地点” (c), “产地 Local” (d), “地点” (e), etc.
2. Non-standardized Formats for Temporal and Spatial Data
Temporal and geographical data lack a universal standard, demanding robust and flexible parsing strategies.
• eventDate: Represented in diverse formats such as “1982年08月24日” (g), “8/10/2008” (b), and “2005.11.10” (c).
• geographical Coordinates: Expressed in various notations, including decimal degrees (“E102.2324946 N23.4191026”, a), and degrees-minutes-seconds (“E99°06′355″ N27°11′628″”, h).
3. Complex Layouts and Structural Heterogeneity
Label layouts often deviate from simple, structured forms, presenting significant challenges for field segmentation and value association.
• Composite Fields with Concatenated Values: Two or more distinct attributes are recorded under a single field label, leading to their values being presented as a contiguous string even without clear delimiters (e.g., recordedBy and recordNumber in d, g, l, n; longitude and latitude in c, e, f; habit and associated species in b, c).
• Multi-field Lines: Multiple, independent fields presented on a single line (e.g., habitat and elevation in a, j; habit and elevation in g, k).
• Multi-line Content: The value of a single field spans across multiple lines (e.g., locality in b, f, i).
4. Handwriting and Image Quality Degradation
Historical specimens introduce fundamental optical challenges that adversely affect OCR accuracy.
• Legible Handwriting: Clear writing enabling successful recognition (l).
• Illegible Handwriting: Scratchy text leading to OCR failure or character misrecognition (p).
• Image Degradation: Physical deterioration of the label, including paper color fading (m), ink bleeding or fading (n, o), low contrast (k), and uneven backgrounds causing OCR failure (j). File: oo_1458163.zip https://binary.pensoft.net/file/1458163 Juan Wen

A3443B85-6102-5B0E-8735-C38AF1E88D9B 10.3897/BDJ.14.e177202.suppl3 Supplementary material 3 Batch Output Results for Image Text Recognition Data type excel table Brief description This supplementary table provides the per-image OCR results generated by DeepSeek LLM on 359 printed-label images, following the exclusion of one image due to complete extraction failure. It serves as the detailed dataset supporting the analysis in the manuscript. File: oo_1502299.xlsx https://binary.pensoft.net/file/1502299 Juan Wen

## Figures and Tables

**Table 1.** Composition of the experimental dataset.

**Institution**	**Training Dataset**			**Control Dataset**		
	Printed	Handwritten	**Total**	Printed	Handwritten	**Total**
PE	120	80	**200**	48	32	**80**
KUN	120	80	**200**	48	32	**80**
IBSC	120	80	**200**	48	32	**80**
**Total**	**360**	**240**	**600**	**144**	**96**	**240**

**Table 2.** Performance comparison between printed and handwritten specimen labels

**Dataset**	**Metric**	**OCR Confidence (per image)**	**OCR Recognition Time (s per image)**	**DeepSeek Extraction Time (s per image)**	**Field Extraction Accuracy (per image)**
**Printed Label**	Mean	0.962	3.28	8.42	0.954
	Std	0.019	0.49	2.17	0.051
	Max	0.991	5.78	41.36	1.000
	Min	0.900	1.50	5.89	0.719
**Handwritten Label**	Mean	0.891	3.52	7.12	0.647
	Std	0.029	0.51	0.86	0.113
	Max	0.950	5.56	14.82	0.885
	Min	0.783	2.03	5.61	0.385
