# Supplementary figures and images for: Automated information extraction from plant specimen labels using OCR and large language models
Source: Biodivers Data J. 2026 Jan 19;14:e177202. doi: 10.3897/BDJ.14.e177202 (PMC12835874; doi:10.3897/BDJ.14.e177202)

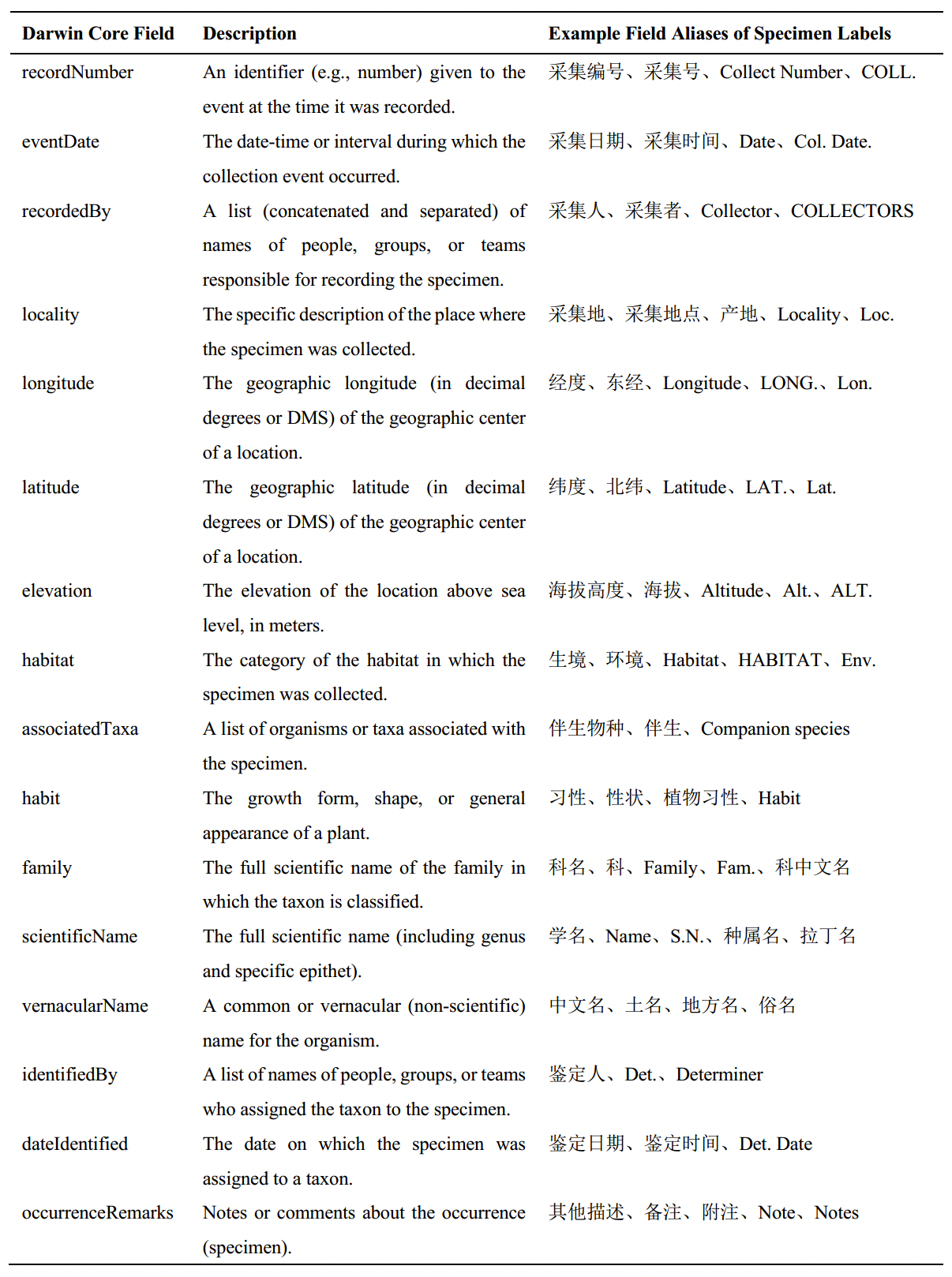

Supplement: Supplementary material 1 — Darwin Core Field Mapping and Descriptions [file bdj-14-e177202-s001.png]

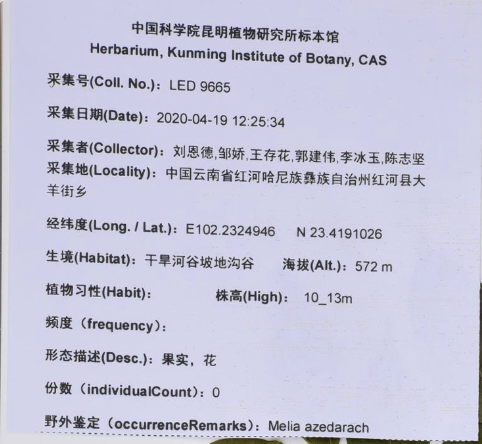

Supplement: Supplementary material 2 — Representative Examples of Label Heterogeneity and Parsing Challenges [file bdj-14-e177202-s002.zip › Suppl. material 2/a.png]

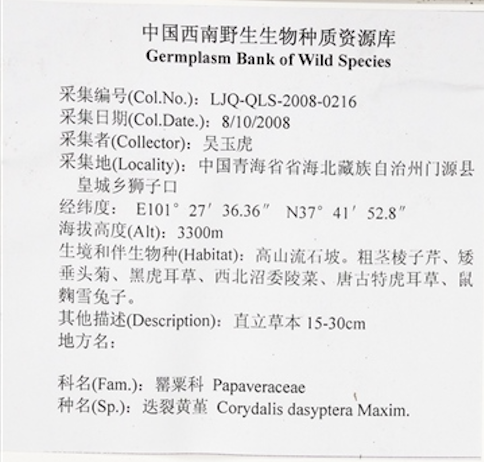

Supplement: Supplementary material 2 — Representative Examples of Label Heterogeneity and Parsing Challenges [file bdj-14-e177202-s002.zip › Suppl. material 2/b.png]

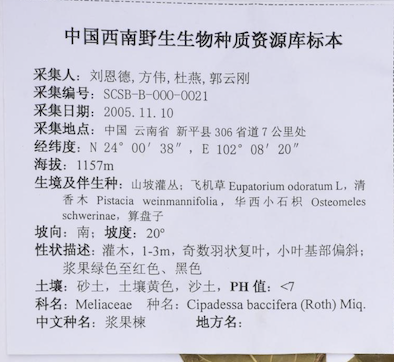

Supplement: Supplementary material 2 — Representative Examples of Label Heterogeneity and Parsing Challenges [file bdj-14-e177202-s002.zip › Suppl. material 2/c.png]

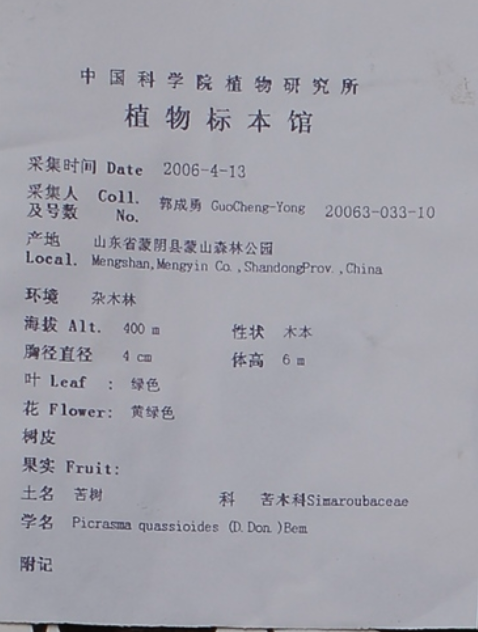

Supplement: Supplementary material 2 — Representative Examples of Label Heterogeneity and Parsing Challenges [file bdj-14-e177202-s002.zip › Suppl. material 2/d.png]

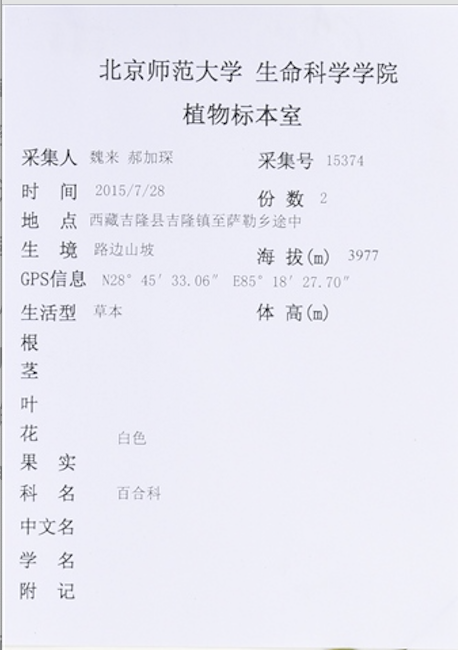

Supplement: Supplementary material 2 — Representative Examples of Label Heterogeneity and Parsing Challenges [file bdj-14-e177202-s002.zip › Suppl. material 2/e.png]

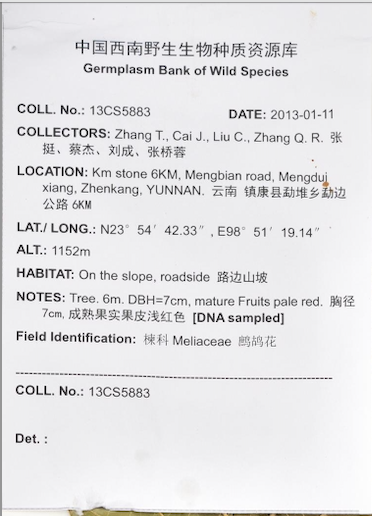

Supplement: Supplementary material 2 — Representative Examples of Label Heterogeneity and Parsing Challenges [file bdj-14-e177202-s002.zip › Suppl. material 2/f.png]

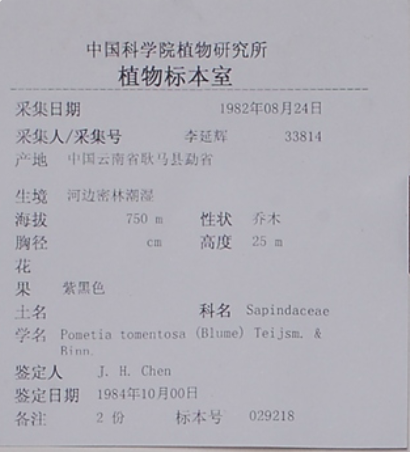

Supplement: Supplementary material 2 — Representative Examples of Label Heterogeneity and Parsing Challenges [file bdj-14-e177202-s002.zip › Suppl. material 2/g.png]

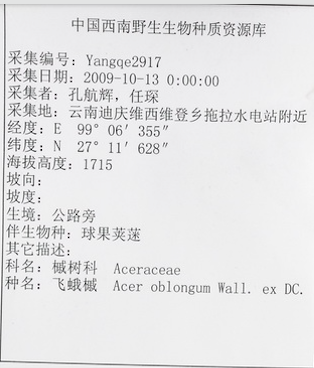

Supplement: Supplementary material 2 — Representative Examples of Label Heterogeneity and Parsing Challenges [file bdj-14-e177202-s002.zip › Suppl. material 2/h.png]

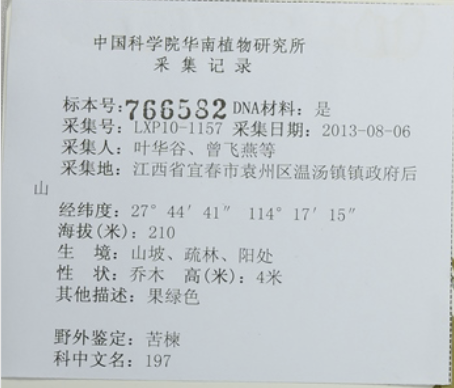

Supplement: Supplementary material 2 — Representative Examples of Label Heterogeneity and Parsing Challenges [file bdj-14-e177202-s002.zip › Suppl. material 2/i.png]

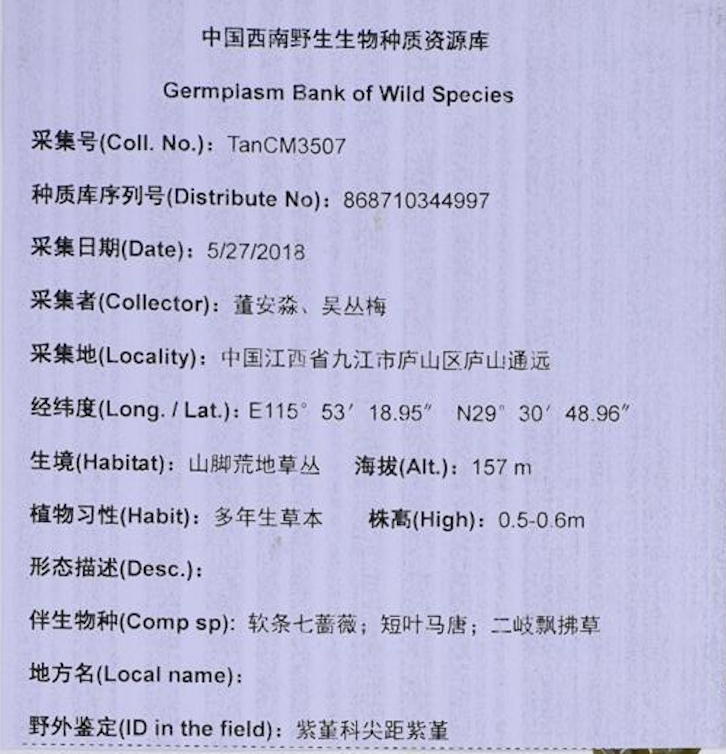

Supplement: Supplementary material 2 — Representative Examples of Label Heterogeneity and Parsing Challenges [file bdj-14-e177202-s002.zip › Suppl. material 2/j.png]

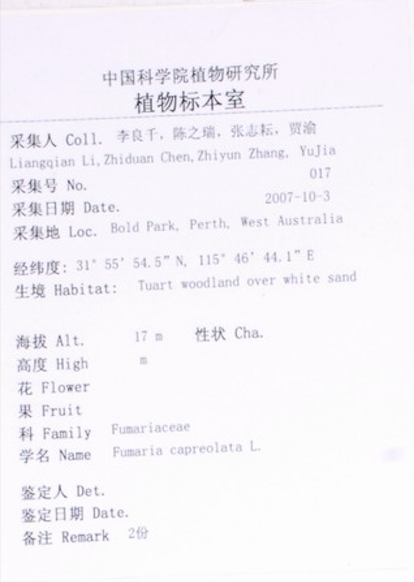

Supplement: Supplementary material 2 — Representative Examples of Label Heterogeneity and Parsing Challenges [file bdj-14-e177202-s002.zip › Suppl. material 2/k.png]

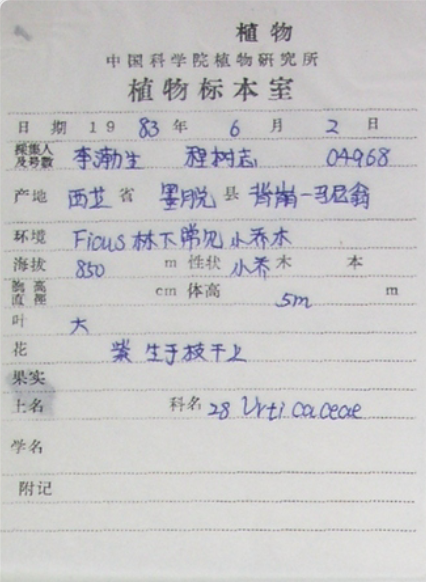

Supplement: Supplementary material 2 — Representative Examples of Label Heterogeneity and Parsing Challenges [file bdj-14-e177202-s002.zip › Suppl. material 2/l.png]

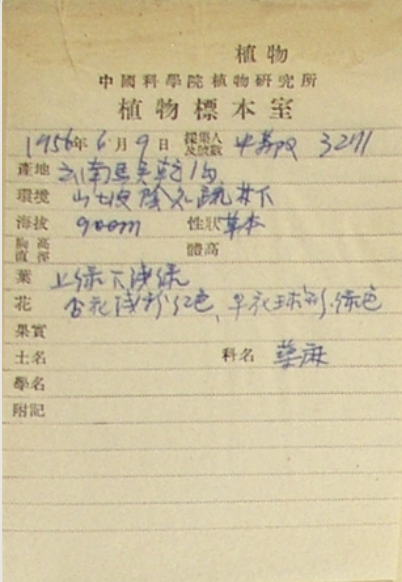

Supplement: Supplementary material 2 — Representative Examples of Label Heterogeneity and Parsing Challenges [file bdj-14-e177202-s002.zip › Suppl. material 2/m.png]

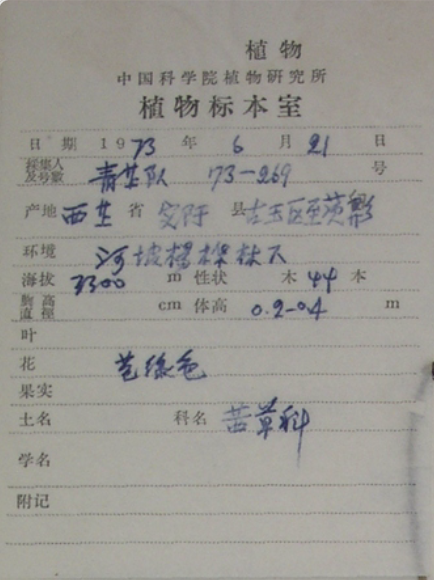

Supplement: Supplementary material 2 — Representative Examples of Label Heterogeneity and Parsing Challenges [file bdj-14-e177202-s002.zip › Suppl. material 2/n.png]

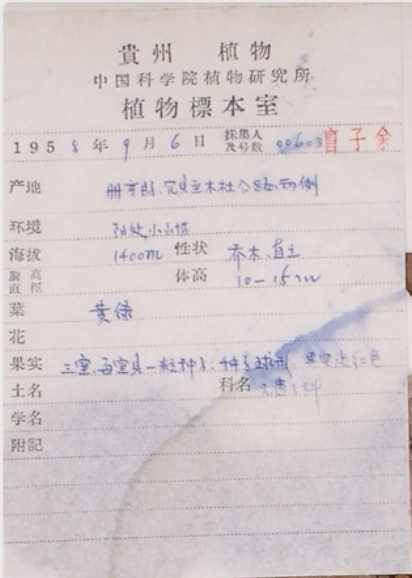

Supplement: Supplementary material 2 — Representative Examples of Label Heterogeneity and Parsing Challenges [file bdj-14-e177202-s002.zip › Suppl. material 2/o.png]

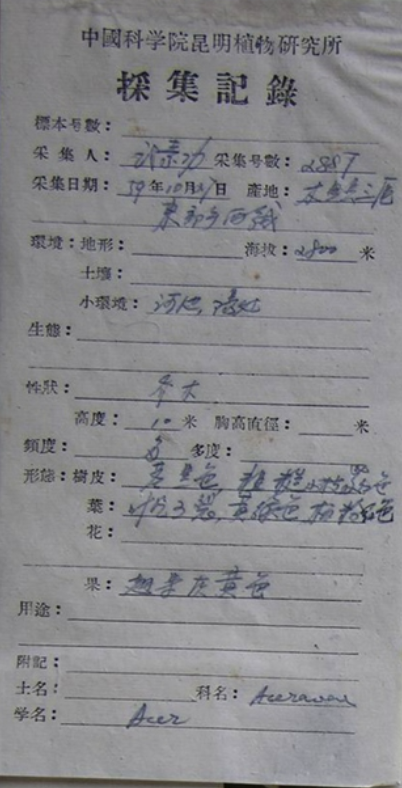

Supplement: Supplementary material 2 — Representative Examples of Label Heterogeneity and Parsing Challenges [file bdj-14-e177202-s002.zip › Suppl. material 2/p.png]
